# The complete mitochondrial genome of *Euplatypus parallelus* (Coleoptera: Curculionidae)

**DOI:** 10.1080/23802359.2016.1275840

**Published:** 2017-04-10

**Authors:** Yan Yang, Xin-Guo Wang, Ya-Xiao Li, Hong-Xia Liu, Qing-Xiang Chai, Zhen-Min Lian, Zhao-Ming Wei

**Affiliations:** a College of Life Sciences, Shaanxi Normal University, Xi’an, China;; b Huangpu Exit-Entry Inspection and Quarantine Bureau Guangzhou, Guangzhou, China

**Keywords:** *Euplatypus parallelus*, mitochondrial genome, Coleoptera

## Abstract

Beetles in the weevil subfamily Platypodinae are among the dominant groups of insects in wet tropical forests, which together with bark and ambrosia beetles in the subfamily Scolytinae. Easily recognised by the circle-shaped entrance holes in fallen logs and a very elongated body shape, they have earned the common name, “pinhole borers”. All except two Platypodinae species are ambrosia beetles that cultivate fungi in wood tunnels as the sole food for their larvae. Platypodinae is a peculiar weevil subfamily of species that cultivate fungi in tunnels excavated in dead wood. The Platypodinae is likely the oldest known lineage of fungus-cultivating insects, with an origin of the ambrosial habit more than 80 Ma. Here, we sequenced and characterized the complete mitochondrial genome of E. parallelus, which was collected from logs imported from SierraLeone. The complete circular mitochondrial genome (mitogenome) of *Euplatypus parallelus* is 16,095 bp in size, containing 37 typical genes and one non-coding AT-rich region. The AT content of the AT-rich region is 87.5%. All protein-coding genes (PCGs) start with standard ATN initiation codons except for *nad1*and end with complete termination codons TAA except for *cox1*genes using an incomplete stop codon T. *tRNA* genes are predicted with a characteristic cloverleaf secondary structure except for *trnS1^(AGN)^*, whose dihydrouridine (DHU) arm is replaced by a simple loop. The size of the large and small ribosomal RNA genes are 1386 and 741 bp, respectively.

Curculionidae is a largest family of Coleoptera with more than 50,000 species worldwide. To date, 1200 species have been recorded in China. Despite this large taxonomic diversity, information about the Curculionidae mitogenomes is still limited. Platypodinae is a peculiar weevil subfamily of the Curculionidae, which has the most prominent economic meaning (Kirkendall & Faccoli 2010). Molecular biological techniques are widely applied to insect systematics (Leavitt et al. 2013). The specimen of *E. parallelus* was collected from logs imported from SierraLeone (About 6 degrees xiking 12 degrees north latitude) in February 2013, was stored in animal laboratory in Shaanxi Normal University with an accession number is 130221, identified by researcher Xin-Guo Wang of Huangpu Exit-Entry Inspection and Quarantine Bureau Guangzhou (Figure 1).

**Figure 1.**
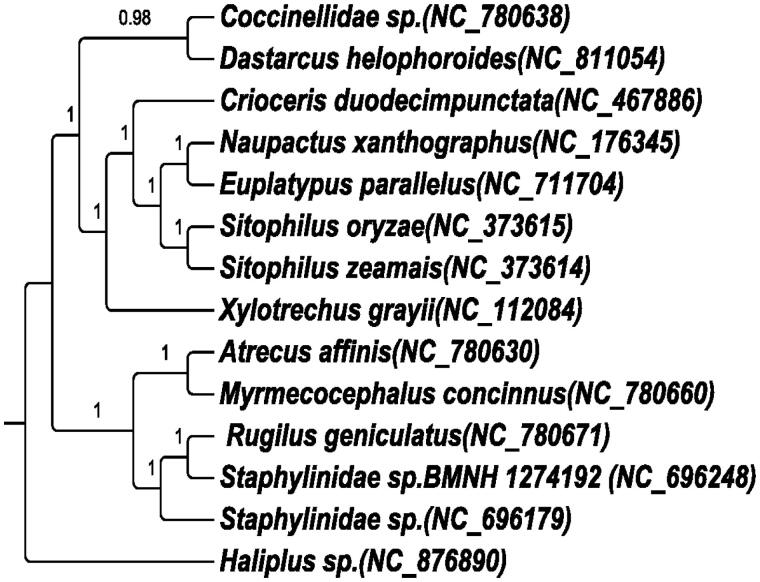
The BI phylogenetic tree of *E. parallelus* based on 13 PCGs dataset. The BI phylogenetic tree of *E. parallelus.* A BI phylogenetic tree was reconstructed using 13 PCGs, GTR + I+G was selected as the best model, and with one Termitidae species as outgroups. Numbers on each node indicate the bootstrap value. Leaf names were presented as species names and Genbank accession number.

The complete mitogenome of *E. parallelus* is a closed circular molecule of 16,095 bp in length, with the typical gene content as other known Coleoptera mitogenomes: 22 transfer RNA genes (tRNAs), 2 ribosomal RNA genes (*rrnL* and *rrnS*), 13 PCGs, and 1 major non-coding AT-rich region (Table 1). Its gene order and orientation are identical to those of other beetles. In addition to the AT-rich region, 11 intergenic spacers (366 bp in total) and 15 overlapping regions (71 bp in total) are dispersed throughout the whole genome. The nucleotide composition of A, T, C, and G are 41.6%, 34.5%, 14.4%, and 9.5%, respectively. The overall nucleotide content is significantly biased toward AT (A + T content =76.1%). Analysis of base composition at each codon position of the concatenated 13 PCGs showed that the first codon position (81.0%) is higher in A + T content than the second (66.8%) and third (73.0%) positions. All PCGs are initiated by a typical ATN codon (five with ATT, three with ATG, and four with ATA), except for four genes (*nad1*). Twelve PCGs have a complete stop codon, while the cox1 gene end with a single nucleotide T. Analysis of 22 tRNA of *E.* parallelus shows the characteristic cloverleaf secondary structure, except for *trnS1^(AGN)^* which lacked the dihydrouridine (DHU) arm and replaced by a single loop. The *rrnL* and *rrnS* of the *E. parallelus* are 1386 bp and 741 bp, respectively. Furthermore, the two rRNAs are also significantly biased towards AT nucleotides (77.60% for rrnL and 77.20% for rrnS). *trnI* locates in AT-rich region and divided into two sections.

**Table 1.** Annotation of mitochondrial genome of *E. parallelus*.

Gene	Direction	Position	Size (bp)	Intergentic Length^a^	Anti codon	Start codon	Stop codon
*trnQ*	R	1–69	69		UUG		
*trnM*	F	69–137	69	1	CAU		
*nad2*	F	138–1145	1008	0		ATT	TAA
*trnW*	F	1145–1199	55	1	UCA		
*trnC*	R	1213–1274	62	−13	GCA		
*trnY*	R	1279–1343	65	−4	GUA		
*cox1*	F	1336–2875	1540	8		ATT	T
*trnL2^*(UUR)*^*	F	2876–2938	63	0	UAA		
*cox2*	F	2939–3619	681	0		ATT	TAA
*trnK*	F	3622–3692	71	−2	CUU		
*trnD*	F	3692–3754	63	1	GUC		
*atp8*	F	3755–3910	156	0		ATT	TAA
*atp6*	F	3907–4578	672	4		ATA	TAA
*cox3*	F	4578–5360	783	1		ATG	TAA
*trnG*	F	5365–5427	63	−4	UCC		
*nad3*	F	5428–5778	351	0		ATT	TAA
*trnA*	F	5777–5837	61	2	UGC		
*trnR*	F	5838–5899	62	0	UCG		
*trnN*	F	5897–5962	66	3	GUU		
*trnS1^*(AGN)*^*	F	5963–6029	67	0	UCU		
*trnE*	F	6029–6092	64	1	UUC		
*trnF*	R	6091–6154	64	2	GAA		
*nad5*	R	6154–7872	1719	1		ATA	TAA
*trnH*	R	7873–7935	63	0	GUG		
*nad4*	R	7935–9260	1326	1		ATA	TAA
*nad4L*	R	9257–9550	294	4		ATA	TAA
*trnT*	F	9557–9619	63	−6	UGU		
*trnP*	R	9620–9683	64	0	UGG		
*nad6*	F	9686–10,192	507	−2		ATT	TAA
*cob*	F	10,196–11,332	1137	−3		ATG	TAA
*trnS2^*(UCN)*^*	F	11,342–11,406	65	−9	UGA		
*nad1*	R	11,433–12,389	957	−26		TTG	TAA
*trnL1^*(CUN)*^*	R	12,391–12,455	65	−1	UAG		
*rrnL*	R	12,416–13,801	1386	40			
*trnV*	R	14,098–14,161	64	−296	UAC		
*rrnS*	R	14,161–14,901	741	1			
AT-rich region		14,902–16,095	1194	0			
*trnI*	F	15,558–15,621	64	64	AAU		

^a^ The positive number indicates interval base pairs between genes, while the negative number indicates the overlaping base pairs between genes.

## Nucleotide sequence accession number

The complete genome sequence of *E.* parallelus has been assigned GenBank accession number KX711704.
